# Essential oil composition and total phenolic content in *Cupressus* *arizonica* G. in response to microbial inoculation under water stress conditions

**DOI:** 10.1038/s41598-023-28107-z

**Published:** 2023-01-21

**Authors:** Hamed Aalipour, Ali Nikbakht, Mohammad R. Sabzalian

**Affiliations:** 1 Department of Horticulture, College of Agriculture, Isfahan University of Technology, Isfahan, 84156-83111 Iran; 2 Department of Agronomy and Plant Breeding, College of Agriculture, Isfahan University of Technology, Isfahan, 84156-83111 Iran

**Keywords:** Microbiology, Plant sciences

## Abstract

Arizona Cypress is one of the drought-resistant, aromatic, and aesthetically pleasing trees having several pharmacological uses. Certain microorganisms contribute to the secondary metabolism and synthesis of bioactive compounds in aromatic and medicinal plants. This study aimed to determine the photosynthetic pigments, total phenolic content, antioxidant capacity, and essential oil composition of Arizona cypress under two irrigation regimes and microbial inoculations. We established a factorial experiment with three mycorrhizae inoculations (*Rhizophagus* *irregularis*, *Funneliformis* *mosseae*, and a mixture of *R.* *irregularis* and *F.* *mosseae*), a rhizobacterium inoculation (*Pseudomonas* *fluorescens*), and two irrigation regimes (well-watered and water stress). Under the water stress regime, seedlings inoculated with *F.* *mosseae* (0.46%) and non-inoculated control plants (0.29%) had the highest and lowest essential oil contents, respectively. GC–MS analysis revealed that limonen, a-pinene, terpinen-4-ol, and umbellulone were the most abundant compounds in the seedlings and treatments under study. The water stress regime had a significant and dominant effect on essential oil and antioxidant capacity, whereas seedling growth and photosynthetic pigments tended to decrease under stress conditions. However, co-inoculation of seedlings with mycorrhizae and the bacterium resulted in an increase in phenolic compounds and carotenoids. Under conditions of water stress and mycorrhizal symbiosis, the results of the current study may help increase the level of valuable compounds in Arizona cypress for further pharmaceutical applications.

## Introduction

Arizona cypress (*Cupressus* *arizonica* Greene) is a drought-tolerant, aromatic, and ornamental tree^1^. It is cultivated in the southern United States as an ornamental and for windbreaks^1^. For therapeutic targets, essential oil (EO) of Arizona cypress can be used as a therapy for broken capillaries and varicose veins, as an astringent and tonic for skin, as an immune system stimulant, and as a sedative^2^. Arizona cypress releases an aromatic scent that is used for various pharmaceutical and cosmetic purposes^2^. Moreover, Arizona cypress has antibacterial and antifungal properties^3,4^. Some compositions of the EO obtained from Arizona cypress could be considered as natural larvicides against *Anopheles* *stephensi* which is a major malaria vector^5^. The most important compounds in the leaf EO of Arizona cypress are p-cymene, a-pinene, cis-muurola-3,5-diene, and germacrene D which account for about 40% of the oil. Other major chemical components are limonene and umbellulone^5^.

As one adverse environmental stressor, drought is a significant concern to overall agricultural production in arid and semi-arid regions, which might even aggravate in the future^6^. In particular, water stress severely inhibits plant growth and finally reduces the overall performance^7^. Morovere environmental factors such as water stress can notably influence the EO yield and composition in aromatic plants^8–10^. Plants exposed to water stress usually generate higher levels of secondary metabolites^11^. The increase of EO yield and its main constituents under water stress in medicinal plants have already been reported in many studies^9,10,12,13^.

The quality and composition of EO might be highly influenced by different environmental factors^14^. Among the biotic factors, using beneficial microorganisms has received serious attention. One such group of microorganisms is arbuscular mycorrhizal fungi (AMF) which can lead to the production of bioactive chemical compounds in medicinal and aromatic plants^15,16^. The inoculation of plants with AMF is one of the most promising tools to alleviate water stress effects in host plants^17^. Other beneficial microorganisms that can increase drought tolerance in host plants and hence the survival of plants under drought conditions are plant growth promoting rhizobacteria (PGPR)^18^. Various researches have also stated that the inoculation of plants with PGPR has beneficial effects on plant growth and crop production^13,19,20^.

Many studies have been undertaken to see synergistic interactions between PGPR and AMF in the various plants^13,20,21^. Previously, synergistic effects of co-inoculation of AMF with PGPR on the composition of bioactive compounds in medicinal plants have also been reported. Hemashenpagam and Selvaraj reported that combined inoculations of *Glomus* *aggregatum* and *Bacillus* *coagulans* with *Trichoderma* *harzianum* led to a significant increase in phenols, flavonoids, alkaloids, saponins, and tannins of *Solanum* *viarum*^22^. Another study by Alam et al. showed enhanced total oil yield in *Pelargonium* *graveolens* co-inoculated with *G.* *intraradices* or/and *G.* *mosseae* with *Bacillus* *subtilis*^23^. Singh et al. have also reported that co-inoculation of symbiotic fungi with PGPR in medicinal plants acts as a useful tool for the improvement of plant essential oils^24^.

In the current study, plant growth, EO composition, total phenolic content, antioxidant capacity, as well as some photosynthetic pigments in Arizona cypress were investigated under two irrigation regimes and microbial inoculations. We hypothesized that one of the factors leading to a reduction in the growth rate of Arizona cypress is water stress. We believe no study investigated the role of AMF and *P.* *fluorescens* inoculation on the qualitative variations and the EO composition of Arizona cypress under water stress conditions. Therefore, the aims of our study were (1) to evaluate the effects of microbial inoculation on biomass, pigment concentrations, total phenolic contents, antioxidant activity, and EO composition of Arizona cypress under well-watered and water stress regimes.; and (2) to evaluate the interaction between AMF and *P.* *fluorescens* in order to improve Arizona cypress tolerance to water stress.

## Material and methods

### Experimental setup

We collected cones from a single Arizona cypress tree grown in Isfahan University of Technology forest (32°39′ N, 51°40′ E; 1590 m). Plant material was collected with the consent of the Urban Landscape Research Group, Isfahan University of Technology. No further legal requirements were necessary for the cone collection of this ornamental tree. The collection of the plant material complied with the relevant institutional (Isfahan University of Technology), national, and international guidelines and legislation. We removed seeds from cones and germinated them in dark (4 °C for 2 weeks) before carefully placing germinants in autoclaved media (120 °C for 2 h) and growing them for six months before transplanting to 10-L pots. Some important characteristics of the original soil are listed in Table 1. These data were obtained from the laboratory of the Department of Soil Science, College of Agriculture, Isfahan University of Technology. Pots were remained in a greenhouse and maintained at 28/16 ± 2 °C day/night and a relative humidity of 30–35% for duration of the study.

**Table 1:** The characteristics of the original soil used in the experiment.

Texture	Sand	Silt	Clay	organic carbon content	CaCO_3_	pH	EC	N	P*	K**	Fe	Zn	Mg
	%	%	%	g Kg^−1^	%		(dS m^−1^)	mg Kg^−1^					
Clay loam	30	41.9	28.1	8.3	35.1	7.72	0.97	30	6.2	120	8.5	0.7	96

*Olsen available.

**Available potassium (Kavail).

A factorial experiment comprised of AMF at four inoculation levels, *P.* *fluorescens* at two inoculation levels, and irrigation regimes at two levels were conducted using a completely randomized design with three replications (three plants in each replication). For mycorrhizal treatments, we inoculated the seedlings with either *Rhizophagus* *irregularis* (Ri) or *Funneliformis* *mosseae* (Fm), or an *R.* *irregularis*–*F.* *mosseae* mixture (Mix). For this, we added 60 g (50–60 spores per g) of mycorrhizal inoculum (National Soil and Water Research Center, Karaj, Iran) to each pot (mixed with the growing media) before transplanting seedlings. Non-inoculated plants served as control. For the PGPR regime, after transplanting seedlings, we applied 4 mL (previous studies indicated that this size of inocula is enough for the amount of soil used) of the *P.* *fluorescens* (Ps) suspension (10^7^ CFU mL^−1^) to the surface of the media in each pot. In addition, we applied 4 mL of sterile distilled water to control (non-inoculated) pots. For the irrigation regime, 5 months after the transplantation, we used either 100% or 50% field capacity, denoted as well-watered (normal watering; N) or water stress (S) treatments, respectively according to the soil–water characteristic curve. Furthermore, fertilization during the plant growth was conducted by the addition of 2 g L^−1^ of commercial fertilizer (20-5-10 N-P-K, 12.8% S, and 1.3% MgO; NovaTec Solub, Compo, Germany) at transplanting and before the start of irrigation regimes.

### Root colonization and seedling biomass

To calculate the root colonization at the end of the experiment, root tissue samples were cleared with KOH (10% w/v) at 95 °C for 55 min. Next, the roots were soaked for 50 min in HCl,. After cutting roots into 10 mm fragments, 100 pieces were placed in trypan blue (0.05% w/v) in lactoglycerol for 20 min. Finally, the gridline intersection method of Giovannetti and Mosse was used for the quantified percentage of root colonization under light microscopy^25^.

We partitioned each seedling into shoot and root after determining fresh weight. Next, for the determination of dry weight, we separately bagged shoots before air-drying. Finally, we dried all samples to a constant weight in anoven at 65 °C before weighting.

### Pigment concentrations

For the determination of photosynthetic pigments (chlorophyll and carotenoid contents), 0.2 g of fresh leaves samples were homogenized in acetone (10 mL, 100%) using a centrifuge run at 4000×*g* for 15 min. Finally, pigment concentrations were determined spectrophotometrically according to the method proposed by Lichtenthaler^26^.

### Determination of total phenolic

In order to measure the total phenolic content, 0.2 mL of the leaf extract was added with Folin-Ciocalteu’s reagent in water (10%, 2.5 mL). Briefly, two grams of the plant aerial parts powder was extracted in 10 mL of 80% methanol for 24 h at 25 °C. An aliquot of 500 µg of the methanolic extract solutions was mixed with 5 mL of tenfold diluted Folin–Ciocalteu reagent (1:10 Folin–Ciocalteu, distilled water) and 4 mL of 7.5% sodium carbonate. Next, samples were kept at room temperature for 60 min, and the absorption rate was measured at a wavelength of 760 nm using a spectrophotometer (UV-160A UV–Visible Recording Spectrophotometer, Shimadzu, Tokyo, Japan)^27^.

### Antioxidant activity

In order to measure the antioxidant activity, 0.2 mL of plant extracts was prepared in methanol (80%) before the 2, 2-diphenyl-1-picrylhydrazyl (DPPH) solution (0.1 mM, 5 mL) was mixed with 200 μL of the diluted extracts. Next, samples were kept in the dark conditions for 30 min at 25 °C. The absorption rate was measured at 517 nm using a spectrophotometric device against a blank containing the methanol solution. Butylated hydroxytoluene was used as the standard antioxidant. Finally, antioxidant activity by scavenging DPPH was calculated using the following equation^28^:

1 $${{\left( {{\text{Blank}}\;{\text{absorbance}} - {\text{Sample}}\;{\text{absorbance}}} \right)} \mathord{\left/ {\vphantom {{\left( {{\text{Blank}}\;{\text{absorbance}} - {\text{Sample}}\;{\text{absorbance}}} \right)} {{\text{Blank}}\;{\text{absorbance}}}}} \right. \kern-0pt} {{\text{Blank}}\;{\text{absorbance}}}} \times 100$$

### Essential oil extraction

The aerial parts (young and mature needles/branches) of the Arizona cypress seedlings (22-month-old) were dried in the shade at room temperature (20 ± 2 °C) for one week, cut into small pieces, then ground using a blender mill. The EO content was extracted using the hydro-distillation method using a Clevenger-type apparatus for 3 h according to what described in Polish Pharmacopoeia VI^29^. The EO was dried over anhydrous sodium sulfate (Na2SO4) and stored in a sealed vial at 0 °C until to be analyzed. EO content (%) was calculated based on the dry matter by the following equation:

2 $${\text{EO}}\;{\text{content}}\;\left( \% \right) \, = \frac{{{\text{mass}}\;{\text{of}}\;{\text{EO}}\;{\text{obtained}}\;\left( {\text{g}} \right)}}{{{\text{mass}}\;{\text{of}}\;{\text{dry}}\;{\text{matter}}\;\left( {\text{g}} \right)}} \times 100$$

### GC–MS analysis and identification of the composition of the essential oils

The EO composition was analyzed using the Agilent 7890 gas chromatographer with an HP- 5MS 5% phenylmethyl siloxane capillary column (30 m × 0.25 mm, and a film thickness of 0.25 μm). The analyses were accomplished using helium as the carrier gas in a split ratio of 1:20 at a flow rate of 2 mL min^−1^. The initial oven temperature was optimized at 60 °C for 3 min and ramped at 3 °C min^−1^ to 120 °C before it was raised to 300 °C at 15 °C min^−1^. The injector temperature was maintained at 300 °C. An Agilent mass selective detector 5975 C was applied in this research. The scanning conditions comprised 39–400 m/z, 200 °C, and an electron ionization of 70 eV. The gas chromatograph (GC) was a Shimadzu GC-17 equipped with an FID detector, and fused-silica column (BP-5, 25 m × 0.22 mm, film thickness 0.25 mm). The operating conditions were: oven temperature 60–280 °C with the rate of 8 °C/min; injector temperature 280 °C, split ratio 1:10, with the carrier gas, N2; detector temperature 300 °C.

EO constituents were identified according to retention indices (RI). The criteria were identified relative to a homologous series of n-alkanes (C5–C24) under the same operating conditions. Further identification was accomplished by comparison of the mass spectra library of compounds^30^.

### Statistical analysis

Data were presented as the mean of three independent assays with respective SD bars (mean ± SD). Data were assessed for normality and, when necessary, log-transformation was used before analysis. Then, data were subjected to a three-way ANOVA analysis. Next, the least significant difference (LSD) test was used to detect specific differences. That the results of the mean comparison were only presented for the traits with statistically significant effects of experimental factors. ANOVA was performed using SAS software (SAS Institute, Cary, NC, USA, 1990). Principal component analysis (PCA) was used to reduce the number of variables measured on Arizona cypress and to measure multivariate co-linearity among the traits and items which was performed using StatGraphics (ver. 9).

## Results

### AM colonization and seedling biomass

Root colonization by AMF significantly decreased in the S condition compared to the N treatment. Interestingly, root colonization increased by Ps regardless of the irrigation regimes (Table 3). The highest root colonization belonged to the mixture of two AMF species (54.7%) in the presence of Ps in the N condition, and the lowest (19.4%) (regardless of non-inoculated treatment) belonged to the treatment of RI species in S condition (Table 3). No fungal colonization was observed on any of the non-mycorrhizal control plants (Table 3).

The biomass of Arizona cypress seedlings (shoot and root dry weights) was significantly affected by AMF inoculation, Ps inoculation, irrigation level, and their interactions (Table 2). Specifically, seedling biomass was adversely affected by the S conditions compared with the N conditions. In contrast, the seedling biomass of plants inoculated with the AMF species significantly increased under the S conditions compared with the non-inoculated controls (Table 3). Moreover, under the N conditions, the largest seedling biomass was found in response to the co-inoculation with Ps and Mix. The results clearly indicated that the co-inoculation of seedlings by AMF and Ps significantly improved Arizona cypress growth performance (Table 3).

**Table 2:** Analysis of variance (including source of variation, degrees of freedom [df], mean square values, P values, and coefficient of variation [CV]) for root colonization (Clon), shoot dry weight (DWS), root dry weight (DWR), total chlorophyll (Chlab) content, carotenoids (Car) content, DPPH activity, total phenolic (Phe) content, and essential oil (EO) content of Arizona cypress seedlings inoculated with two arbuscular mycorrhizae (*Rhizophagus* *irregularis* and *Funneliformis* *mosseae*) and one rhizobacterium (*Pseudomonas* *fluorescens*) grown under two irrigation regimes.

Source of variation	df	Clon	DWS	DWR	Chlab	Car	DPPH	Phe	EO
Irrigation regime (A)	1	876**	5.66**	5.68**	13.0**	0.902**	164**	7.53*	0.0052**
Mycorrhiza inoculation (B)	3	3676**	1.05**	1.05**	0.768*	0.955**	453**	120**	0.0046**
*Pseudomonas* inoculation (C)	1	1988**	1.85**	1.17**	0.099^ ns^	3.88**	21.2^ ns^	10.0**	0.00003^ ns^
A × B	3	133**	0.203*	0.184*	0.431*	0.229*	26.2*	2.04^ ns^	0.0033**
A × C	1	295**	0.414**	0.259*	1.22**	1.27**	33.5*	10.9**	0.0005^ ns^
B × C	3	230**	0.401**	0.286*	0.137^ ns^	0.167*	12.8^ ns^	4.65*	0.0132**
A × B × C	3	50.2**	0.273**	0.225*	0.102^ ns^	0.432**	5.83^ ns^	2.72^ ns^	0.0053**
Error	32	8.29	0.049	0.045	0.014	0.025	0.740	1.13	0.0004
CV (%)		11.1	11.2	12.9	10.2	12.1	5.50	12.8	5.09

*ns* not significant.

*Significant at the 5% probability level.

**Significant at the 1% probability level.

**Table 3:** Interaction effects of irrigation regime × AMF × PGPR on root colonization (Clon), shoot dry weight (DWS), root dry weight (DWR), DPPH activity, and total phenolic (Phe) of Arizona cypress seedling.

Treatments	Clon (%)	DWS (g plant^−1^)	DWR (g plant^−1^)	DPPH (%)	Phe (mg TAE g^−1^ DW)
N					
C	0.01 ± 0.0^h^	3.60 ± 0.10^c^	2.66 ± 0.11^cd^	39.1 ± 2.0^i^	4.4 ± 0.21^h^
RI	24.3 ± 3.0^ef^	3.38 ± 0.19^cd^	2.50 ± 0.07^d^	43.0 ± 2.0^hi^	6.1 ± 1.6^e–h^
FM	23.6 ± 1.6^fg^	3.19 ± 0.10^c–f^	2.29 ± 0.48^de^	48.6 ± 2.5^fg^	7.2 ± 0.44^e^
Mix	30.7 ± 4.4^cd^	3.25 ± 0.63^c–f^	2.58 ± 0.49^cd^	43.4 ± 4.7^hi^	5.0 ± 0.90^gh^
Ps	0.01 ± 0.0^h^	3.41 ± 0.12^e^	2.28 ± 0.12^de^	45.1 ± 3.3^gh^	5.2 ± 1.5^f–h^
RI + Ps	43.1 ± 2.5^b^	4.35 ± 0.10^b^	3.06 ± 0.15^bc^	49.7 ± 1.0^ef^	9.2 ± 1.0^cd^
FM + Ps	42.4 ± 4.7^b^	4.52 ± 0.23^b^	3.35 ± 0.25^b^	56.5 ± 2.1^a–c^	10.4 ± 1.4^bc^
Mix + Ps	54.7 ± 3.2^a^	4.71 ± 0.18^a^	3.97 ± 0.11^a^	51.9 ± 5.6^d–f^	10.8 ± 0.24^bc^
S					
C	0.01 ± 0.0^h^	1.73 ± 0.07^g^	0.983 ± 0.21^f^	43.4 ± 2.4^hi^	4.7 ± 0.62^gh^
RI	19.4 ± 2.3^g^	2.86 ± 0.09^d–f^	1.91 ± 0.09^e^	50.8 ± 2.1^d–f^	6.8 ± 1.4^ef^
FM	21.1 ± 2.7^fg^	3.06 ± 0.19^c–f^	2.16 ± 0.15^de^	57.3 ± 2.0^ab^	7.8 ± 0.92^de^
Mix	24.5 ± 1.7^ef^	2.72 ± 0.06^f^	1.92 ± 0.06^e^	54.5 ± 1.1^b–d^	6.3 ± 1.1^e–g^
Ps	0.01 ± 0.0^h^	1.90 ± 0.14^g^	1.03 ± 0.04^f^	42.5 ± 3.0^hi^	7.7 ± 1.1^de^
RI + Ps	35.9 ± 2.4^c^	3.17 ± 0.06^c–f^	2.31 ± 0.06^de^	53.3 ± 1.8^b–e^	11.6 ± 1.1^ab^
FM + Ps	28.5 ± 4.7^de^	2.83 ± 0.09^e–f^	1.97 ± 0.14^e^	60.1 ± 2.0^a^	12.8 ± 1.2^a^
Mix + Ps	32.8 ± 4.2^cd^	2.49 ± 0.32^c^	2.46 ± 0.21^d^	52.4 ± 1.3^c–f^	12.1 ± 0.71^ab^

Means (± SD) followed by similar letters within each column do not express signifiant diffrences according to LSD at *P* < 0.05.

N well watered (100% field capacity); S water stress (50% field capacity), RI *Rhizophagus irregularis*, FM *Funneliformis mosseae*, Mix: *R. irregularis*–*F. mosseae* mixture, C non-inoculated plants, Ps *Pseudomonas fluorescens*, TAE tannic acid equivalent.

### Photosynthetic pigments

**Figure 1:**
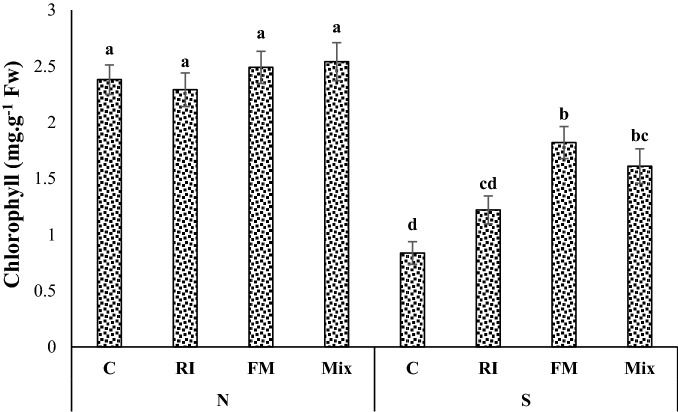
Interactions of irrigation regime × mycorrhizae on chlorophyll contents of Arizona cypress seedlings. N well watered (100% field capacity), S water stress (50% field capacity), RI *Rhizophagus irregularis*, FM *Funneliformis mosseae*; Mix: *R. irregularis*–*F. mosseae* mixture; C: Non-inoculated plants.

**Figure 2:**
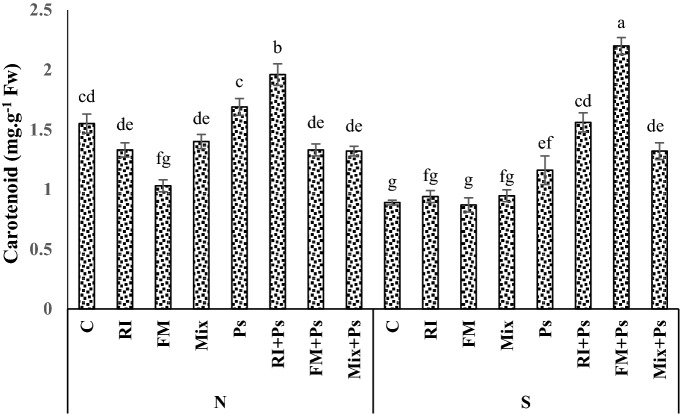
Interactions of irrigation regime × mycorrhizae × rhizobacterium on carotenoid contents of Arizona cypress seedlings. N well watered (100% field capacity); S: water stress (50% field capacity); RI *Rhizophagus irregularis*, FM *Funneliformis mosseae*; Mix *R. irregularis*–*F. mosseae* mixture, C non-inoculated plants, Ps *Pseudomonas fluorescens*.

Under the S regime, chlorophyll and carotenoids decreased while an increase in chlorophyll and carotenoids was observed in the leaves of inoculated seedlings compared to non-inoculated plants (Figs. 1 and 2). The Arizona cypress seedlings inoculated with FM and Mix increased contents of chlorophyll under S conditions compared to the non-inoculated control plants (Fig. 1). In seedlings subjected to S, AMF colonization by FM led to the maintenance of a much higher chlorophyll content than in non-inoculated seedlings (Fig. 1). Also, under S conditions, co-inoculation of AMF with Ps significantly increased carotenoid content of Arizona cypress seedlings compared to the non-inoculated control plants (Fig. 2). Especially, co-inoculation by FM and Ps was shown to be more effective in improving carotenoid content than other treatments under S regime (Fig. 2).

### Phenolic content and antioxidant activity

The total phenolic content (Phe) of Arizona cypress seedlings significantly decreased under the S regime compared to the N regime, and a similar trend was also observed for the antioxidant activity (Table 3). Regardless of the irrigation regimes, the lowest Phe content was found in non-inoculated plants. Furthermore, co-inoculation of all AMF species with *P.* *fluorescens* significantly increased the amounts of Phe compared with single inoculations (Table 3).

The antioxidant activity was significantly influenced by irrigation regimes and AMF inoculation (Table 2). A higher antioxidant activity was obtained from seedlings inoculated with FM and Ps compared with non-inoculated controls regardless of the irrigation regimes (Table 3). Neither RI nor Mix inoculation was useful for mitigating the effects of S in terms of the increase in antioxidant activity. Co-inoculated plants with FM and Ps showed the highest antioxidant activity, suggesting the synergistic effects of FM and Ps on this characteristic (Table 3).

### Essential oil content and composition

EO yield showed low variation under different treatments. It ranged from 0.29% in non-inoculated control plants to 0.46% in *F.* *mosseae* alone inoculated seedlings under the water-deficit regime. Under the N regime, the lowest and the highest EO contents were identified in non-inoculated control plants (0.33%) and the treatment of *R.* *irregularis–F.* *mosseae* mixture with *P.* *fluorescens* inoculation (0.44%), respectively (Table 4).

**Table 4:** Essential oil composition of Arizona cypress seedlings inoculated with two arbuscular mycorrhizae (*Rhizophagus* *irregularis* and/or *Funneliformis* *mosseae*) and one rhizobacterium (*Pseudomonas* *fluorescens* alone or in combination with AMF) grown under two irrigation regimes.

Treatments	EO (%)	α-thujene	α-pinen	Myrcene	Camphene	1-Octen-3-ol	Sabinene	p-cymene	γ-terpinen	Terpinolene	Nonan-2-one	Umbellulone
N												
C	0.33^hi^	1.5	14	0.9	3.8	17.2	3.6	3.3	3.5	1.1	3.6	7.0
RI	0.42^bc^	1.6	13.5	0.7	3.0	15.2	3.7	3.9	3.9	1.3	4.0	7.5
FM	0.41^b–e^	1.7	13.6	0.7	3.4	16.9	3.0	3.3	3.8	1.5	3.4	7.2
Mix	0.35^gh^	1.5	12.8	0.9	3.9	17.2	3.1	3.5	3.7	2.0	3.7	7.5
Ps	0.38^e–g^	1.3	13.4	1.1	4.1	17	2.8	3.6	3.6	1.2	3.9	7.3
RI + Ps	0.40^c–f^	1.5	13.3	0.8	3.5	15.9	3.2	3.5	3.7	1.4	3.9	7.2
FM + Ps	0.37^fg^	1.4	13.3	0.9	3.1	16.5	3.0	3.6	4.1	1.6	3.7	7.5
Mix + Ps	0.44^ab^	1.4	12.9	0.8	3.2	16.9	3.7	3.2	3.7	1.3	3.8	7.9
S												
C	0.29^i^	1.1	14.4	0.7	3.5	15	3.2	3.1	4.2	1.2	4.1	6.9
RI	0.37^fg^	1.4	14.4	1.2	3.6	16.6	3.4	3.3	3.8	1.3	4.1	7.6
FM	0.46^a^	1.3	13.6	0.4	3.3	15.2	3.4	3.4	4.4	1.4	4.9	7.3
Mix	0.38^e–g^	1.6	15.5	0.5	3.4	17.9	3.2	2.9	3.7	1.6	3.8	8.1
Ps	0.32^hi^	0.9	14.5	0.6	2.7	15.3	3.8	3.4	3.8	1.9	4.3	7.4
RI + Ps	0.39^d–f^	1.6	13	1.0	3.5	16	2.8	3.7	3.3	1.5	4.4	7.3
FM + Ps	0.35^gh^	1.6	13.9	0.6	3.2	15.5	3.4	3.6	4.1	1.6	4.6	8.1
Mix + Ps	0.42^bc^	1.3	14.6	0.7	3.8	17.8	2.8	2.7	3.3	1.3	3.7	8.4
RI		925	936	995	953	958	975	1026	1064	1085	1093	1170

Treatments	Terpinen-4-ol	α-ylangene	α-copaene	Aromadendrene	Allo-aromadendrene	γ-muurolene	γ-curcumene	Bicyclogermacrene	α-muurolene	γ-cadinene	Germacrene D	Cedrol
N												
C	7.2	1.3	3.6	2.4	1.6	0.7	0.8	3.1	2.9	2.5	4.4	2.8
RI	6.6	1.6	4.5	2.3	2.2	0.6	0.6	3.6	3.5	2.6	4.3	2.4
FM	6.8	1.3	3.8	2.4	2.1	0.8	0.7	3.2	2.9	3.0	4.0	2.6
Mix	7.4	1.7	4.2	2.1	1.6	0.4	0.7	3.0	2.7	2.7	4.2	2.7
Ps	6.6	1.5	3.8	2.6	1.3	0.9	0.5	2.8	3.3	2.7	4.6	2.6
RI + Ps	6.8	1.7	4.4	2.2	2.4	0.6	0.7	3.7	3.3	2.4	4.4	2.6
FM + Ps	6.9	1.6	3.8	2.2	1.9	0.7	0.7	3.4	3.0	2.9	3.8	2.9
Mix + Ps	7.6	1.6	4.1	2.0	1.5	0.5	0.6	3.4	2.9	2.8	4.4	2.3
S												
C	7.0	1.7	4.3	2.2	1.7	0.8	0.3	3.2	3.0	2.6	4.0	2.5
RI	6.7	1.3	4.1	2.2	1.5	0.5	0.3	3.6	2.9	2.6	4.5	2.7
FM	6.8	1.7	4.4	2.4	1.9	0.5	0.7	3.9	3.1	2.3	3.7	2.6
Mix	7.1	1.2	4.2	1.9	1.7	0.3	0.5	3.4	2.6	2.8	4.7	2.5
Ps	7.4	1.8	4.3	2.5	1.8	0.4	0.2	2.2	3.1	2.9	4.2	2.1
RI + Ps	6.6	1.4	4.4	2.5	1.7	0.5	0.3	3.7	3.2	2.8	4.8	2.4
FM + Ps	6.9	1.9	4.6	2.8	2.4	0.2	0.4	3.5	2.8	2.1	4.0	2.2
Mix + Ps	8.3	1.1	3.9	2.4	1.6	0.7	0.6	3.5	2.3	2.5	4.5	2.9
RI	1182	1373	1378	1441	1456	1474	1480	1491	1501	1515	1556	1596

Within each column means followed by the same letters do not express significant differences according to LSD at *P* < 0.05.

N well watered (100% field capacity), S water stress (50% field capacity), RI *Rhizophagus irregularis*, FM *Funneliformis mosseae*, Mix *R. irregularis* mixture, C Non-inoculated plants, Ps *Pseudomonas fluorescens*.

Twenty three compounds were identified as the main components in the EO profile of Arizona cypress among which, the four components of limonen (15–17.9%), a-pinene (12.8–15.8%), terpinen-4-ol (6.6–8.3%), and umbellulone (6.9- 8.3%) were identified as the major ones. While different types of microorganisms had different effects on each of these compounds, among the AMF species, the highest amounts of limonen and a-pinene belonged to the Mix inoculation under the S regimes (Table 4). Co-inoculated plants with Mix and Ps recorded higher amounts of terpinen-4-ol and umbellulone under the S regimes. Some components rose in quantity in non-inoculated plants under N regimes rather than under the S regimes. For example, α-thujene, camphene, γ-curcumene, and cedrol increased under the N regime compared to S one. In contrast, some components were reduced when the seedlings were not inoculated under the N regimes rather than the S regimes. The highest quantities of p-cymene and germacrene D belonged to the treatment of RI (3.9%) and the co-inoculation of RI with Ps (4.8%), respectively. Sabinene and terpinolene recorded their highest amounts in the individuals inoculated with Ps. terpinolene and umbellulone production decreased in non-inoculated plants. It shows that application of beneficial microorganisms was able to stimulate the production of some special EO compositions (Table 4).

### Principal component analysis (PCA)

**Figure 3:**
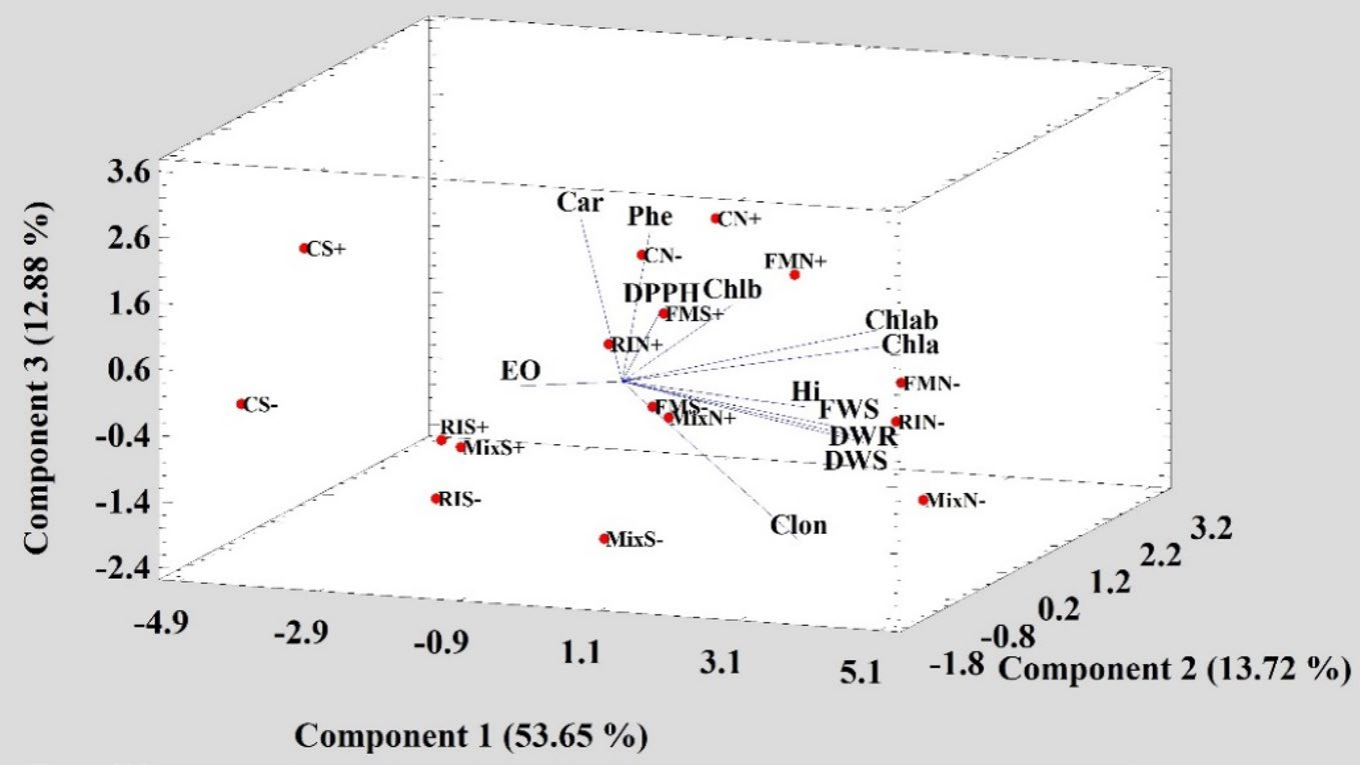
Principal component analysis (PCA) was performed on characteristics of Arizona cypress inoculated with *Rhizophagus* *irregularis*, *Funneliformis* *mosseae* and a mixture of both species with and without *Pseudomonas fluorescens* inoculation. N well watered (100% field capacity), S: water stress (50% field capacity), RI *Rhizophagus* *irregularis*, FM *Funneliformis* *mosseae*, Mix *R.* *irregularis*–*F.* *mosseae* mixture, +  *Pseudomonas* *fluorescens* inoculation, − without *Pseudomonas* *fluorescens* inoculation, C non-inoculated plants, Hi: Plant height, FWS shoot fresh weight, DWS shoot dry weight, DWR root dry weight, EO essential oil content, Phe total phenolic contents, Clon root colonization, Chla Chlorophyll *a*, Chlb chlorophyll *b*, Chlab total chlorophyll, Car Carotenoids.

PCA analysis was performed to measure multivariate co-linearity among the traits, to group treatments applied and to choose the best treatments regarding different characteristics. The triplot results show that the three principal components explained 80.25% of the variability (Fig. 3). PC1 had positive coefficients for all characteristics, excluding DPPH, Phe, carotenoid (Car), and EO. It seems that this PC could be considered as morphology and chlorophyll index. On the contrary, PC2 had positive coefficients for DPPH, and Phe and PC3 had a high positive coefficient for Car. Therefore, these two latter components are more representative of antioxidant properties indices.

According to the triplot chart, seedling growth parameters had high and positive correlations with the percentage of root colonization by both AMF species. While EO had a negative correlation with all the characteristics, including the rate of colonization. Also, the antioxidant capacity of seedling extracts was not influenced by EO content and AMF colonization (Fig. 3).

The triplot clearly shows that all treatments under water stress (S) were separated entirely from those under well-watered treatments (N), indicating the highly discriminating effect of irrigation regimes over microbial inoculations. The combination of irrigation regimes and microbial treatments showed dispersal in all dimensions. In the N zones where PC1 values are highly positive, MixN− and RiN− are the best treatments regarding higher seedling growth while in S zones, where the PC1 is negative, FMS+, and FMS− were the best treatments regarding higher PC2 values and antioxidant activity. Therefore, it seems that after the water deficit, AMF inoculation has a higher discriminating value over Ps inoculation. Also, as observed in the triplot, CS+, and CS− both are the weakest treatments fully separated from the other treatments (Fig. 3).

## Discussion

In our study, highly significant effect of AMF colonization was observed in seedlings co-inoculated with AMF and Ps compared to those inoculated only with AMF. The results confirmed past results of other researchers that reported a higher amount of AMF colonization in co-inoculated plants as compared to individual inoculation^13,20,31,32^. This observation could be attributed to the presence of PGPR, which may act as a ‘mycorrhization helper bacteria’, and in so doing they result in the better establishment of mycorrhizae in the host root and better function of mycorrhizae^32,33^.

The results showed that Arizona cypress seedlings inoculated with different AMF species would make higher chlorophyll and carotenoid contents than non-inoculated plants under both N and S conditions. Our previous study has also revealed that different AMF species increased the total chlorophyll and carotenoid contents of Arizona cypress^20^. Terpenoids are involved in plant growth, development, and responses to environmental stresses such as drought^12^. Terpenoids are also involved in plant responses against pathogens^34^. Isoprenoid metabolites such as chlorophyll, carotenoids, and terpenoids are important for the acclimation and adaptive responses of plants to environmental stresses‏^35^. In the present investigation, higher amounts of photosynthetic pigments present in all inoculated plants compared to control plants might be the key factor for the improvement of Arizona cypress growth under water stress conditions. Carotenoid content may also improve the antioxidant capacity of seedlings grown under water stress conditions.

In this experiment, antioxidant activities rose in the AMF inoculated seedlings which can be explained by the presence of high phenolic contents, which are well known for the strong antioxidant activity^36^. AMF inoculation also had a much higher effect on antioxidant capacity compared to Ps inoculation. However, co-inoculation with both AMF and Ps increased the antioxidant properties. Eftekhari et al. reported an elevation in Phe as a result of inoculation by AMF in *Vitis* *vinifera*^37^. Mechri et al. also showed a similar trend, showing that the Phe in olive trees rose when plants were inoculated with mycorrhizal species^38^. Dutta and Neog reported that co-inoculation of *Curcuma* *longa* L. with AMF and PGPR provided a higher Phe content than non-inoculated plants^31^. AMF could accumulate phenylalanine ammonia-lyase, which is the key enzyme for the biosynthesis of phenolic compounds^39^.

In this study, the EO content of seedlings was not affected by AMF and/or Ps colonization. It may be originated from a short time when seedling were under the influence of inoculations. Also, it seems that water stress treatment dominated microbial inoculations in this regard. However, the interaction of beneficial microorganisms with medicinal plants has been reported as an important factor that helps plants to protect themselves from stresses^40^. Urcoviche et al. found that the EO content of *Mentha* *crispa* L. inoculated with AMF increased from 0.2% to 0.98% when compared with non-inoculated plants^41^. Fokom et al. in the same plant reported that inoculation with *G.* *etunicatum* showed the highest EO content compared to non-inoculated plants^16^.

EO is produced and stored in leaf structures of medicinal plant known as peltate glandular^42^. AMF-inoculated plants could alter the number of peltate trichomes in medicinal plants, and improve some EO components^43^. Also, some microorganisms could change the concentration of EO due to the change in plant hormones. Gibberellins and cytokines for example has been proposed to be involved in changing the EO concentrations^44,45^.

Rahimzadeh and Pirzad also reported that higher oil and mucilage contents of flaxseed (*Linum* *usitatissimum* L.) plants were obtained from co-inoculated plants with mycorrhizae and PGPR compared with non-inoculated plants, suggesting the synergistic effects of AMF and PGPR^13^. However, the mechanisms behind the effects of beneficial microorganisms on EO components are not clear but probably it may be related to the better absorption of nutrients^15^.

The present study demonstrates that in all treatments, limonen, a-pinene, terpinen-4-ol, and umbellulone were the major components. The influence of microbial inoculation on the EO composition of Arizona cypress, varied according to the different microorganisms. For example, the highest amounts of terpinen-4-ol and umbellulone belonged to the co-inoculated plants with mixed AMF strains and Ps under water stress regimes (Table 4). A greenhouse study on *Thymus* *daenensis* also showed enhancement in concentrations of thymol, when plants were inoculated with a mixture of *G.* *mosseae* and *P.* *fluorescens*^46^. Synergistic effects of three different AMF, including *Glomus*, *Gigaspora*, *Acaulospora* sp. with three PGPR; *Bacillus* *megaterium*, *Azospirillum* *amazonense*, and *Azotobacter* sp.; also showed an increase in flavonoids, phenolic and curcumin contents in *Curcuma* *longa*^31^. Vafadar et al. found that the application of *G.* *intraradices* in combination with *Bacillus* *polymixa* or/and *Pseudomonas* *putida* increased stevioside content compared to single inoculated or non-inoculated plants^47^. In our study, however, the effect of water stress treatment was more discriminating as a-pinene, and umbellulone were more accumulated after water stress treatment.

Probably the effects of beneficial microorganisms on EO composition are due to changes in the EO biosynthesis^15^. The essential oils are synthesized by two different biochemical pathways; phenylpropanoids were derived from the shikimic acid pathway, and isoprenoids/terpenoids were derived from the 2-C-methyl-d-erythritol 4-phosphate pathway. Thus, changes in the EO composition would be due to the impacts of microorganisms on the products of both pathways^48^.

Microorganisms can improve plant nutritional status when soil water is limited by increasing the uptake of nitrogen (N), phosphorus (P), and other nutrients that can increase the EO content and their specific compounds in aromatic plants^49,50^. For example, N plays a critical role in the synthesis of many plant natural components such as amino acids and enzymes that play key roles in the biosynthesis of the EO composition^50^. Also, medicinal plants need acetyl CoA, ATP and NADPH for the biosynthesis of terpenoids, and the availability of these coenzymes is related to the P concentration; hence, improving the absorption of P by plants helps to increase essential oil production^51^.

## Conclusion

In conclusion, this study provided clear evidence that the Arizona cypress seedlings were affected by water stress and microbial agents (AMF and Ps); however, the order of effect was as: S > AMF > PS. Furthermore, the interaction of Arizona cypress with both AMF and Ps in co-inoculation did not result in improved plant biomass and essential oil composition as compared to individuals inoculated only with AMF. It seems that AMF alone could induce the reprogramming of many metabolic products like phenolic compounds and DPPH in Arizona cypress leading to higher biomass and enhanced antioxidant activity; however, these two characteristics may not be correlated and be AMF inoculation-specific. The use of these beneficial microorganisms under water stress regimes presents a useful option for increased tolerance to the water stress and higher plant growth and metabolites accumulation.

## Data Availability

All data generated and analyzed during this study are included in this paper.

## Abbreviations

AMF: Arbuscular mycorrhizal fungi
PGPR: Plant growth promoting rhizobacteria
ANOVA: Analysis of variance
EO: Essential oil
S: Water stress
RI: *Rhizophagus* *irregularis*
FM: *Funneliformis* *mosseae*
Mix: *R.* *irregularis*–*F.* *mosseae* *mixture*
Ps: *Pseudomonas* *fluorescens*
N: Normal watering (well-watered)
PCA: Principal component analysis
Phe: Total phenolic content
DPPH: 2, 2-Diphenyl-1-picrylhydrazyl
